# High Bioavailability Resveratrol Delivery System: A Novel Nutritional Strategy for the Prevention and Alleviation of Rheumatoid Arthritis

**DOI:** 10.1002/fsn3.71464

**Published:** 2026-02-04

**Authors:** Chenchen Yu, Chungang Zhang

**Affiliations:** ^1^ College of Pharmacy Liaoning University of Traditional Chinese Medicine Dalian China; ^2^ School of Life and Health Dalian University Dalian China

**Keywords:** bioavailability, resveratrol, rheumatoid arthritis, solid dispersion

## Abstract

This study aimed to develop an oral solid dispersion nutrient delivery system of resveratrol (RSV) and Eudragit E PO (E PO) for the prevention of rheumatoid arthritis. The RSV‐E PO solid dispersion, prepared by the solvent method at a drug—polymer ratio of 1:7 (w/w), turned resveratrol into an amorphous state, as proved by SEM, DSC, XRD, and FTIR. Over 80% of resveratrol was released in vitro, a 13‐fold increase compared to raw resveratrol. In male Sprague—Dawley rats, its oral administration (20 mg·kg^−1^) doubled bioavailability versus unformulated resveratrol. Evaluated in an adjuvant‐induced arthritis (AIA) model, the compound demonstrated significant anti‐arthritic effects. These protective effects were primarily mediated through the modulation of key inflammatory and oxidative stress pathways, as evidenced by a marked reduction in pro‐inflammatory cytokines (IL‐6, TNF‐α, IL‐1β) and malondialdehyde (MDA) levels, coupled with an increase in the anti‐inflammatory cytokine IL‐10 and the antioxidant enzyme superoxide dismutase (SOD). Also, its safety was confirmed by stable AST, ALT, CREA, and BUN levels. In summary, the RSV‐E PO solid dispersion, with better dissolution and bioavailability, serves as an effective oral nutrient delivery system for RSV.

## Graphical abstract

This study developed a high bioavailability resveratrol delivery system for rheumatoid arthritis prevention using solid dispersion. Its oral administration (20 mg·kg^−1^) doubled bioavailability versus unformulated resveratrol, and the serum biomarkers related to liver and kidney function did not increase, indicating that the preparation has good safety.

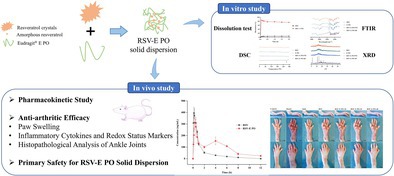

## 1 Introduction

Rheumatoid arthritis (RA) is an autoimmune disease marked by progressive, symmetric joint inflammation resulting in cartilage destruction, bone erosion, and disability (Lin et al. 2020). The prevalence of RA varies across different regions, with higher incidence rates commonly found in industrialized countries (Peng et al. 2024). RA affects approximately 0.5%–1% of the adult population, with a significantly higher prevalence in women than in men (Baig et al. 2024). The initial clinical manifestations of RA include the gradual onset of joint swelling, pain, and stiffness, which may subsequently evolve into symmetrical polyarthritis (Refaat et al. 2013). If left untreated, RA can result in disability and is associated with common extra‐articular manifestations affecting the cardiovascular, pulmonary, ocular, cutaneous, and vascular systems (Zhao et al. 2021).

The development of RA is a complex and protracted process, involving a multifaceted interplay of genetic, environmental, and immunological factors that contribute to immune system dysregulation (Di Matteo et al. 2023). Present pharmacological strategies for RA, such as nonsteroidal anti‐inflammatory drugs, disease‐modifying anti‐rheumatic drugs, biological agents, and glucocorticoids, primarily manage symptoms rather than offering a cure (Zewail et al. 2019). Compounding this limitation, their use is frequently constrained by a profile of significant adverse effects, including systemic toxicity, which remains a major clinical concern (Ali et al. 2023).

Given these deficiencies, there is an urgent need for safer, more effective, and affordable nutraceutical formulation designed for the prevention and alleviation of RA. Emerging evidence and reports revealed that several extracts, monomers from plants and foods were demonstrated the promising anti‐arthritic activity. Natural products have recently gained attention for their low toxicity and minimal side effects. Resveratrol (RSV), a polyphenolic compound (3,5,4′‐trihydroxystilbene) is derived from botanical sources such as grapes, berries, and *Polygonum cuspidatum* and is commonly used in functional foods. It is renowned for its potential health benefits, including antioxidant and anti‐inflammatory properties, anti‐obesity, anticancer, and antibacterial properties (Tian and Liu 2020). Notably, its potential anti‐arthritic activity has also become a focal point of research. A substantial number of studies have demonstrated that resveratrol can effectively inhibit the apoptosis of fibroblast—like synovial cells in rheumatoid arthritis rats compared to arthritis control rats (Wang et al. 2019). It also significantly reduces the levels of serum malondialdehyde (MDA), interleukin‐6 (IL‐6), tumor necrosis factor α (TNF‐α), interleukin—1β (IL‐1β), and the arthritis index score in these animals (Yang et al. 2018). Moreover, a recent randomized controlled clinical trial demonstrated that the clinical and biochemical indices of rheumatoid arthritis in the Resveratrol—treated group were considerably lower than those in the control group (Khojah et al. 2018). It is well established that for an orally administered bioactive nutrient to exert its therapeutic effect, it must first dissolve in the gastrointestinal fluids to become available for absorption across the intestinal membrane and subsequently reach the systemic circulation. However, the bioavailability of RSV is notoriously low, primarily due to its hydrophobic nature, which has been a major obstacle to its practical application. We hypothesize that improving the solubility and bioavailability of RSV via our delivery system is the key mechanism underlying its enhanced prophylactic and therapeutic effects against adjuvant‐induced arthritis. As a result, it is important to select suitable bioactive delivery within the food and pharmaceutical industries. In recent years, various delivery systems of resveratrol have been developed, such as encapsulation strategies, liposomal carriers, emulsion‐based systems, and nano‐targeted technology. However, their widespread application is hampered by complex preparation processes, high production costs, and difficulties in scaling up. Additionally, nano‐targeted formulations cannot be industrialized.

Compared to these systems, solid dispersions are more suitable for industrialization. It has been reported that a total of 48 drug products incorporating amorphous solid dispersions (ASDs) were approved by the U.S. Food and Drug Administration between 2012 and 2023 (Moseson et al. 2024). This indicated that ASD‐based formulations represented a well‐established and clinically viable strategy for enhancing the delivery of poorly water‐soluble drugs. The preparation methods for solid dispersions, such as spray drying and hot‐melt extrusion, are relatively straightforward and readily scalable. Moreover, quality control of solid dispersions is facilitated by their generally favorable stability and improved batch‐to‐batch reproducibility.

Eudragit E PO (E PO), a cationic polyelectrolyte, belongs to the family of (meth)acrylate copolymers. It is composed of dimethylaminoethyl methacrylate, butyl methacrylate, and methyl methacrylate in a molar ratio of 2:1:1 (Li et al. 2015). Widely employed in the pharmaceutical industry, E PO serves multiple functions, including taste masking, moisture protection, release modification, and use as an excipient (Hofmann et al. 2025). Furthermore, it has demonstrated significant potential as a polymeric carrier in solid dispersions for enhancing the dissolution rate of poorly water‐soluble active pharmaceutical ingredients. Therefore, this study aimed to develop a high bioavailability resveratrol delivery system for rheumatoid arthritis prevention using solid dispersion. The RSV‐E PO solid dispersion was developed through the solution method and was characterized using FTIR, DSC, XRD, and release studies at pH 1.2. The anti‐arthritic activity was evaluated using paw swelling volume measurements, histopathological analysis, cytokine level evaluation, and antioxidant biomarker assessment in an adjuvant‐induced arthritis (AIA) model. Furthermore, a preliminary safety assessment was conducted by evaluating renal and hepatic functions.

## 2 Materials and Methods

### 2.1 Materials

Resveratrol (98% purity) was sourced from Wuhan Yuancheng Gongchuang Technology Co. Ltd. (Hubei, China). Carbamazepine (purity of 98%) chosen as internal standard was obtained from Shanghai Haling Biotechnology Co. Ltd. (Shanghai, China). Indomethacin was obtained from Guangdong South China Pharmaceutical Group Co. Ltd. (Guangdong, China). Eudragit E PO was supplied by Evonik Industries AG in Darmstadt, Germany. Methanol and acetonitrile were supplied from OCEANPAK (Sweden). Complete Freund's adjuvant (CFA) was provided from Sigma Aldrich, USA. ELISA kits for rat IL‐6, IL‐10, IL‐1β and TNF‐α were sourced from Thermo Fisher Scientific Biology Science and Technology Co. Ltd. SOD and MDA kits were procured from Nanjing Jiancheng Bioengineering Institute (Nanjing, China). Rat alanine aminotransferase (ALT), aspartate aminotransferase (AST), urea and creatinine kits were obtained from Rayto Life and Analytical Sciences Co. Ltd. (Shenzhen, China).

### 2.2 Animals

The experiments received approval from the Animal Ethics Committee of Liaoning University of Traditional Chinese Medicine (IACUC Issue No. 210000620240226) and were conducted in compliance with laboratory animal care guidelines. Specific pathogen‐free male Sprague–Dawley rats (250 ± 20 g) and Wistar rats (180 ± 20 g) were obtained from Liaoning Changsheng Biotechnology Co. Ltd. (Liaoning, China). Male Sprague–Dawley rats were selected for the pharmacokinetic study due to their well‐established status as a standard model in drug disposition research, facilitating data comparison with the existing literature. Conversely, male Wistar rats were employed for the pharmacodynamic (anti‐arthritic) evaluation because of their high sensitivity and reproducible inflammatory response in the complete Freund's adjuvant‐induced arthritis model. Following a one‐week acclimatization period, the animals were housed under controlled conditions (temperature: 23°C ± 2°C, humidity: 55% ± 5%, 12‐h light–dark cycle) and had free access to standard chow and water throughout the experiment.

### 2.3 Preparation of RSV‐E PO Solid Dispersion

RSV and E PO (1:7 w/w) were homogeneously mixed using a mortar and pestle, then dissolved in ethanol under ultrasonic conditions until a clear solution was achieved. The resulting dispersion was allowed to solidify at room temperature, then ground and sieved through a 60‐mesh sieve. The resulting solid dispersion was finally stored in a light‐protected, dry environment for future use.

### 2.4 In Vitro Dissolution Test

Dissolution tests were performed using the paddle method, following the guidelines of the Chinese Pharmacopeia. The ZRS‐8GD dissolution apparatus from Tianda Tianfa Technology Co. Ltd. in Tianjin, China, was utilized. RSV and RSV‐E PO solid dispersion, each containing 300 mg of RSV, were added to 900 mL of pH 1.2 solution maintained at 37°C ± 0.5°C with a paddle speed of 100 rpm. At designated time intervals, 5 mL samples were taken and replaced with an equal volume of fresh pH 1.2 solution. Subsequently, the samples were then filtered through a 0.45 μm membrane. The resulting filtrate was diluted as needed to prepare the sample solution, which was analyzed using a UV1600 spectrometer from Shanghai Meipuda Instrument Co. Ltd. at a wavelength of 307 nm. All measurements were performed in triplicate, and the results are reported as the mean ± standard deviation (SD).

### 2.5 Characterization of Solid Dispersions

#### 2.5.1 Fourier Transform Infrared Spectroscopy (FTIR)

FTIR spectroscopy was conducted using a FTIR‐850 Fourier transform infrared spectrometer which was produced by Tianjin Gangdong Technology Co. Ltd. in Tianjin, China. Spectra were obtained in the range of 4000 cm^−1^ to 400 cm^−1^ with a resolution of 4 cm^−1^, and each sample underwent 32 scans. The above samples were prepared via KBr disk method.

#### 2.5.2 Differential Scanning Calorimetry (DSC)

Thermograms of the samples were recorded using the DSC 1 STAR instrument (Mettler‐Toledo Inc., Zurich, Switzerland). Approximately 5 mg of each sample was weighed and conducted, with an empty crucible serving as the reference. The samples were heated from 0°C to 300°C at a rate of 10°C·min^−1^.

#### 2.5.3 X‐Ray Diffraction (XRD)

The XRD patterns of solid samples were analyzed using a DX‐2700 powder X‐ray diffractometer from Dandong Haoyuan Instruments Co. Ltd. in Dandong, China. Samples were scanned over a 2*θ* range of between 4° and 60° with a step of 0.03°, operating at a voltage of 40 kV and a current of 25 mA.

### 2.6 Pharmacokinetic Study

#### 2.6.1 Administration Regimen and Sample Collection

Twelve rats were randomized into two groups (*n* = 6 per group) following an overnight fast with ad libitum access to water. RSV or RSV‐E PO solid dispersion was suspended in 0.5% w/v CMC‐Na. Each rat was administered a single intragastric dose of either resveratrol or RSV‐E PO solid dispersion (20 mg resveratrol·kg^−1^). Blood samples (approximately 500 μL) were collected via the orbital vein at specified time points: 0, 0.0833, 0.1667, 0.25, 0.5, 0.75, 1, 2, 4, 6, 8, and 12 h after dosing. Plasma samples were subjected to centrifugation at 12,000 r·min^−1^ for 10 min and then kept at −80°C until analysis.

#### 2.6.2 Sample Preparation and HPLC Assay

Plasma samples were processed using acetonitrile precipitation. Specifically, a total of 100 μL of plasma was combined with 10 μL of methanol, 10 μL of an internal standard solution containing 25 μg·mL^−1^ carbamazepine in methanol, and 50 μL of 0.5% acetic acid. After vortexing for 1 min, 200 μL of acetonitrile was introduced and the mixture was vortexed an additional minute. Subsequently, the mixture was centrifuged at 12,000 r·min^−1^ for 10 min. A 30 μL supernatant aliquot was injected into the HPLC system for analysis.

RSV concentrations in assay samples were tested using a Shimadzu 2010A HPLC system (Kyoto, Japan). Separation was performed using an Agilent Extend C18 column (150 mm × 4.6 mm, 5 μm) at 30°C. The mobile phase consisted of 30% acetonitrile and 70% water, and it was operated at a flow rate of 1 mL·min^−1^. Detection of resveratrol was performed at a UV wavelength of 307 nm.

The calibration curve exhibited linearity (*R* ^2^ > 0.99) within the 10–1000 ng·mL^−1^ concentration range. The lower limit of quantitation (LLOQ) was established at 10 ng·mL^−1^. The relative standard deviations (RSDs) for precision at three different quality control (QC) levels (20, 100, and 800 ng·mL^−1^) were 2.88%, 2.15%, and 1.68% (*n* = 6) for intra‐assay precision, and 2.72%, 2.04%, and 1.98% (*n* = 3) for inter‐assay precision. The recoveries of resveratrol were (99.2% ± 1.3%), (98.6% ± 1.4%), and (101.1% ± 2.1%), respectively. The recovery of the internal standard was (98.7% ± 1.7%).

### 2.7 Anti‐Arthritic Efficacy of the RSV‐E PO Solid Dispersion

#### 2.7.1 Induction of Arthritis and Drug Administration

The AIA model was created by injecting 0.1 mL of CFA containing 1 mg·mL^−1^ *Mycobacterium tuberculosis* into the right hind paw of rats, excluding the control group. Prior to arthritis induction, 42 Wistar rats were randomly divided into seven groups, with six rats in each group. Table 1 summarizes the specific group details and oral dose levels. Figure 2A illustrates the experimental schedule.

**TABLE 1:** Groups and doses of oral administration of AIA rats.

Groups	Dose (mg· kg^−1^)	Mode of administration
Control	Distilled water	Orally given an equal volume of 0.1% CMC‐Na solution
Model	Distilled water	
Indo	5	The drug was suspended in 0.1% CMC‐Na solution and treated by intragastric administration
RSV	20	
RSV‐E PO‐10	Equivalent to 10 mg· kg^−1^ of resveratrol	
RSV‐E PO‐20	Equivalent to 20 mg· kg^−1^ of resveratrol	
RSV‐E PO‐40	Equivalent to 40 mg· kg^−1^ of resveratrol	

#### 2.7.2 Paw Swelling

Rat paw edema indicated arthritic progression. Prior to model induction, baseline measurements of paw dimensions were established. Throughout the experiment, the thickness (H) and width (W) of the right hind paw were assessed every 3 days (3, 6, 9, 12, 15, 18, 21 days) post‐modeling using an electronic vernier caliper (Deli Group Co. Ltd.), recorded in millimeters (mm). The method was implemented with reference to a prior study (Mahdi et al. 2018) and was subsequently optimized to meet the objectives of the present study. Briefly, paw swelling was quantified by calculating the cross‐sectional area, determined as the product of thickness and width. Consistent measurement positioning was ensured during each assessment, with both sides of the vernier caliper touching the paw surface. Each paw was measured three times, and the mean value was recorded.

(1) $$\mathrm{Paw}\;\text{edema}\;\left({\mathrm{mm}}^{2}\right)=W\times H$$

#### 2.7.3 Evaluation of Pro‐Inflammatory and Antioxidant Biomarkers

On the last day of the experiment, rats were anesthetized via intraperitoneal injection of 20% urethane at a dosage of 5 mL·kg^−1^. Blood samples were obtained from the abdominal aorta and centrifuged at 3500 rpm for 10 min after a 30‐min incubation period to separate the serum. The serum was stored at −80°C for later analysis. Serum levels of pro‐inflammatory biomarkers (IL‐1β, IL‐6, TNF‐α) and oxidative stress markers (SOD, MDA) were quantified using ELISA kits. All assays were conducted by a blinded operator according to the manufacturer's protocols.

#### 2.7.4 Histopathological Analysis of Ankle Joints

At the experiment's conclusion, all rats were anesthetized and sacrificed following serum collection. The right ankle joints were immediately dissected, hair and epidermis removed, and fixed in 4% paraformaldehyde. Paraffin‐embedded sections of joint tissues were dewaxed with xylene, rehydrated with a series of ethanol solutions, and stained with hematoxylin–eosin (HE). After dehydration and sealing, the sections were examined under a microscope to assess histopathological alterations in each experimental group.

### 2.8 Evaluation of Primary Safety for RSV‐E PO Solid Dispersion

#### 2.8.1 Determination of Body Weight and Organ Index

Throughout the experiment, the body weight of each rat was monitored every 3 days. After euthanasia, the heart, liver, lungs, kidneys, spleen, and thymus were excised, rinsed with ice‐cold saline, and weighed. Organ indices were calculated based on these measurements at the conclusion of the study.

(2) $$\text{Organ index}=\text{the weight of organ}\;\left(\mathrm{mg}\right)/\text{the body weight of}\;\mathrm{rat}\;\left(g\right)$$

#### 2.8.2 Biochemical Analysis

AST, ALT, urea, and creatinine levels were quantified using commercially available kits with an autoanalyzer, according to the manufacturer's instructions, to assess liver and kidney function in rats.

#### 2.8.3 Histopathological Analysis of Stomach

Since Eudragit E PO is a linear cationic polymer with solubility restricted to acidic aqueous environments, a study was performed to assess the impact of orally administering RSV‐E PO solid dispersion on rat stomachs. After administering the RSV‐E PO solid dispersion orally, rat stomachs were fixed in 4% paraformaldehyde, embedded, sectioned, HE‐stained, dehydrated, and sealed. Microscopic examination was conducted to observe pathological changes.

### 2.9 Statistical Analysis

The data are expressed as mean ± standard deviation (SD). One‐way ANOVA was performed using SPSS 16.0 (SPSS Inc., Chicago, IL, USA) to identify significant differences among groups. A *p*‐value below 0.05 was deemed statistically significant. Blood concentration data for resveratrol were analyzed using DAS 3.0 software (Chinese Mathematics and Pharmacology Professional Committee, Shanghai, China).

## 3 Results

### 3.1 Characterization of Solid Dispersions

#### 3.1.1 FTIR Analysis

**FIGURE 1:**
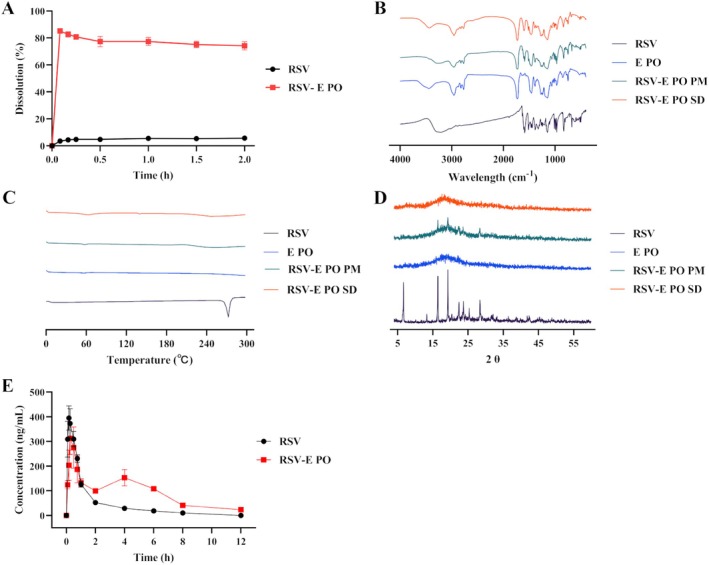
(A) Dissolution profiles of RSV and RSV‐E PO. (B) FTIR spectra of RSV, E PO, the physical mixture of two materials, and the RSV‐E PO solid dispersion. (C) DSC curves of RSV, E PO, the physical mixture of two materials, and the RSV‐E PO solid dispersion. (D) XRD patterns of RSV, E PO, the physical mixture of two materials, and the RSV‐E PO solid dispersion. (E) Plasma concentration‐time profiles of RSV and the RSV‐E PO solid dispersion.

FTIR spectroscopy was used to examine the interaction between RSV and E PO by analyzing RSV, E PO, their physical mixtures, and RSV‐E PO solid dispersion. As illustrated in Figure 1B, crystalline RSV exhibited a characteristic peak at 3250 cm^−1^, which was attributed to the phenolic hydroxyl groups. For E PO, the carbonyl peak appeared at 1730 cm^−1^, accompanied by a broad peak near 3500 cm^−1^, indicating water presence within the polymer matrix. The unchanged carbonyl peak in the RSV‐E PO solid dispersion indicated no significant interaction between RSV and E PO.

#### 3.1.2 DSC Analysis

The physical state of RSV within the solid dispersion was characterized using DSC. Figure 1C demonstrated that raw RSV displayed a distinct endothermic peak at 272°C, reflecting its crystalline structure. Conversely, in the physical mixture, the melting peak of RSV became broader, indicating a gradual dissolution of the drug into the polymer matrix upon heating.

#### 3.1.3 XRD Analysis

The XRD analysis was employed to assess the solid‐state characteristics of the formulations. As shown in Figure 1D, raw RSV displayed prominent diffraction peaks between 4° and 60°, which were also present in the physical mixtures, indicating that RSV maintained its crystalline structure. In contrast, the XRD patterns of the RSV‐E PO solid dispersion revealed halo patterns, suggesting that resveratrol was primarily in an amorphous state within this formulation.

### 3.2 In Vitro Dissolution Test

The dissolution profiles of raw RSV and the RSV‐E PO solid dispersion were compared in Figure 1A. Under the experimental conditions, RSV demonstrated a slow dissolution rate, with less than 10% released after 2 h under the given experimental conditions. In contrast, the RSV‐E PO solid dispersion exhibited significantly improved dissolution, reaching maximum release within 10 min, in contrast to raw RSV. More than 80% of RSV was released from the solid dispersion, representing approximately a 13‐fold enhancement compared to raw RSV. However, the RSV‐E PO solid dispersion did not maintain the maximum release percentage over time.

### 3.3 Pharmacokinetic Study

The oral bioavailability of the RSV‐E PO solid dispersion was assessed in a rat pharmacokinetic study. Figure 1E presented the mean plasma concentration‐time profiles, and the corresponding pharmacokinetic parameters were summarized in Table 2.

**TABLE 2:** Pharmacokinetic parameters of trans‐resveratrol in rat plasma after oral administration of raw trans‐resveratrol and solid dispersions.

Parameters		Resveratrol	RSV‐E PO
AUC_(0–12h)_	ng·mL·h^−1^	538.7 ± 50.1	1120.9 ± 99.0
AUC_(0–∞)_	ng·mL·h^−1^	553.0 ± 49.1	1223.3 ± 116.2
*t*_1/2_	h	2.7 ± 0.35	2.9 ± 0.33
*T*_max_	h	0.17 ± 0.05	0.28 ± 0.11
*C*_max_	ng·mL^−1^	405.2 ± 43.4	353.8 ± 44.7

Following oral administration of raw resveratrol (20 mg·kg^−1^), the drug was rapidly absorbed, reaching a peak plasma concentration (*C*_max_) of 405.2 ± 43.4 ng·mL^−1^ at 0.17 ± 0.05 h (*T*_max_), followed by a decline with a terminal half‐life (*t*_1/2_) of 2.7 ± 0.35 h. The area under the curve from 0 to 12 h (AUC_(0–12h)_) was 538.7 ± 50.1 ng·mL^−1^·h. In contrast, the RSV‐E PO solid dispersion markedly increased the systemic exposure, evidenced by a 2.08‐fold higher AUC_(0–12h)_ (1120.9 ± 99.0 ng·mL^−1^·h). Interestingly, the *C*_max_ (353.8 ± 44.7 ng·mL^−1^) and *T*_max_ (0.28 ± 0.11 h) of the solid dispersion did not show a significant enhancement. Furthermore, the plasma profile of the solid dispersion exhibited a distinct double‐peak phenomenon.

### 3.4 Effect of RSV‐E PO Solid Dispersion on AIA Rat Model

#### 3.4.1 Effect of RSV‐E PO Solid Dispersion on Paw Swelling

**FIGURE 2:**
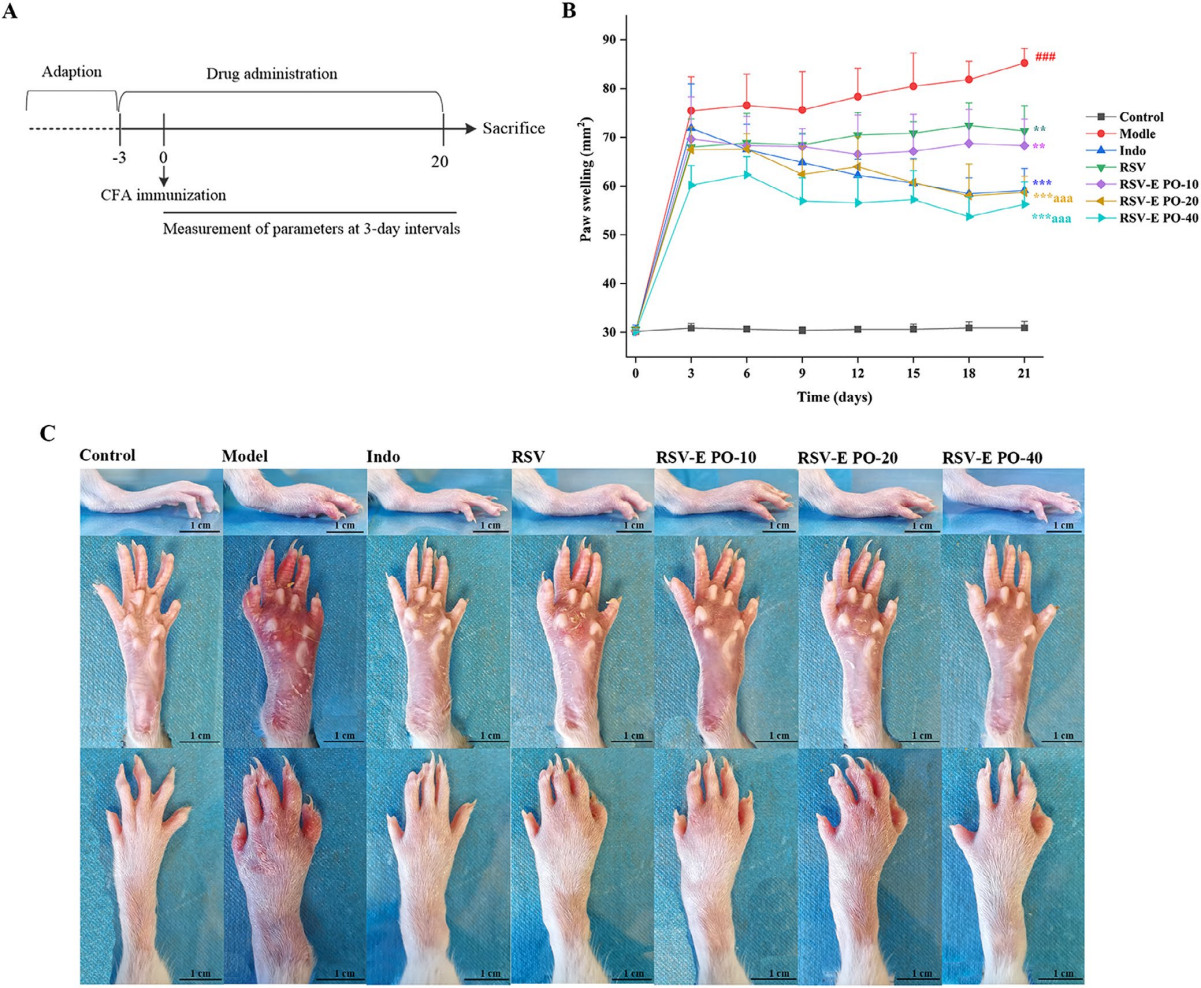
(A) Detailed experimental schedule. (B) Serial measurements of paw volume in different groups. (C) Photographs of the paws of representative rats in different groups. Data are represented as mean ± SD (*n* = 6). ###*p* < 0.001 vs. control group; **p < 0.01, ***p < 0.001 vs. model group; ^aaa^ *p* < 0.001 vs. RSV group.

The therapeutic effects on CFA‐induced arthritis were assessed macroscopically (Figure 2). While CFA injection induced severe paw swelling, stiffness, and mild ulceration in the model group, these pathological features were visibly reduced in all pretreatment groups. Administration of indomethacin, raw RSV, or RSV‐E PO solid dispersion alleviated joint swelling without causing ulceration. A dose‐dependent improvement was observed with the RSV‐E PO formulation, as evidenced by the paw and ankle joints in the middle‐ and high‐dose groups appearing nearly normal.

Paw edema was quantified to evaluate therapeutic efficacy (Figure 2C). The model group developed significant swelling from Day 3 (*p* < 0.01 vs. control), which was ameliorated by all treatments. In particular, the 20 and 40 mg·kg^−1^ doses of the RSV‐E PO solid dispersion were significantly more effective than raw RSV (*p* < 0.05). Furthermore, a significant difference was noted between these two doses on Days 6 and 12 (*p* < 0.05). In contrast, the effects of indomethacin and low‐dose RSV‐E PO became significant at later time points.

#### 3.4.2 Effect of RSV‐E PO Solid Dispersion on Inflammatory Markers

**FIGURE 3:**
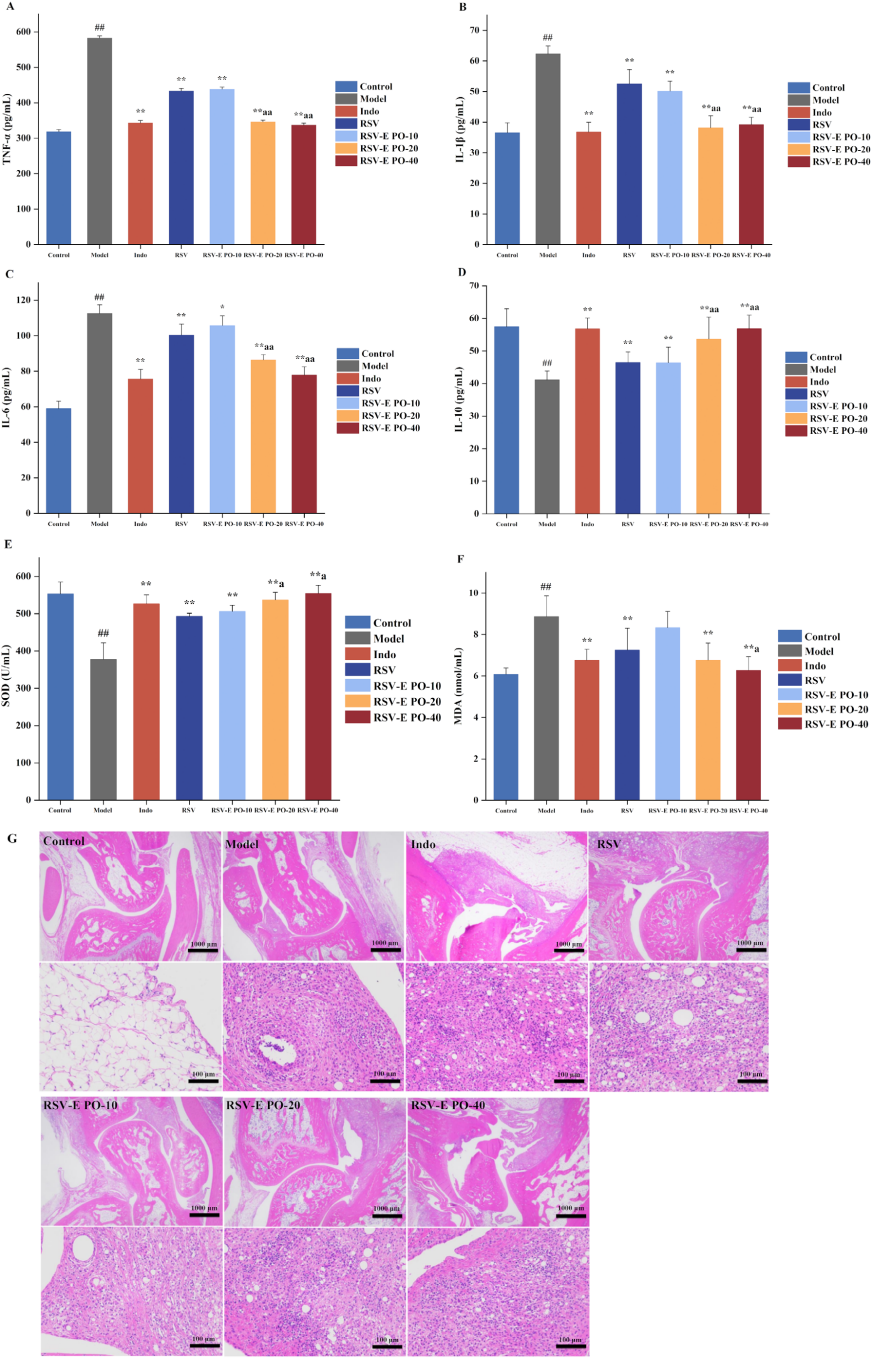
Effects of RSV and RSV‐E PO solid dispersion on (A–D) serum cytokines, (E) SOD, and (F) MDA in adjuvant‐induced arthritis rats and (G) histopathological analysis of ankle joint tissues stained with H&E. Data are represented as mean ± SD (*n* = 6). ##*p* < 0.01 vs. control group; *p < 0.05, **p < 0.01 vs. model group; ^a^ *p* < 0.05, ^aa^ *p* < 0.01 vs. RSV group.

As shown in Figure 3A–D, serum levels of the pro‐inflammatory cytokines TNF‐α, IL‐1β, and IL‐6 were significantly elevated in the model group, while the anti‐inflammatory cytokine IL‐10 was markedly reduced compared to the control group (*p* < 0.01). All treatment groups—Indo, RSV, and the three doses of RSV‐E PO solid dispersion—significantly reversed these changes, suppressing the pro‐inflammatory cytokines and elevating IL‐10 levels relative to the model group (*p* < 0.01). There were also notable differences among RSV, RSV‐E PO‐20, and RSV‐E PO‐40 groups (*p* < 0.01).

#### 3.4.3 Effect of RSV‐E PO Solid Dispersion on Oxidation Indices

CFA injection into the rats' right hind paw significantly increased MDA levels (*p* < 0.01) and notably decreased SOD levels (*p* < 0.01) in comparison to the normal group. Pretreatment with indomethacin, RSV, and various doses of RSV‐E PO solid dispersion significantly increased SOD levels (*p* < 0.01) and significantly decreased serum MDA levels (*p* < 0.01), except in the RSV‐E PO‐10 group. Compared to the resveratrol group, rats in the medium and high dose resveratrol solid dispersion groups had significantly higher serum SOD levels (*p* < 0.05), while the high dose group also exhibited significantly lower serum MDA levels (*p* < 0.05).

#### 3.4.4 Histopathological Impact of RSV‐E PO Solid Dispersion on Joint Tissue

The histological examination of rat ankle joints following HE staining was presented in Figure 3G. In the normal group, the joint tissue exhibited uniform staining, with smooth articular cartilage surfaces and normal morphology and structure of chondrocytes. Additionally, the synovial membranes on both sides showed no significant hyperplasia, and inflammation was absent. In contrast, the model group displayed pronounced thickening of the synovial membrane, characterized by connective tissue hyperplasia, increased infiltration of lymphocytes and neutrophils, and the presence of cavity structures. RSV‐E PO solid dispersion pretreatment improved synovial hyperplasia and reduced lymphocyte and neutrophil infiltration. Notably, the RSV‐E PO‐40 group exhibited a significant decrease in lymphocyte and neutrophil infiltration, along with a marked reduction in cavity structures.

### 3.5 Primary Safety Evaluation of RSV‐E PO Solid Dispersion

#### 3.5.1 Impact of RES‐E PO Solid Dispersion on Body Weight and Organ Indices

**FIGURE 4:**
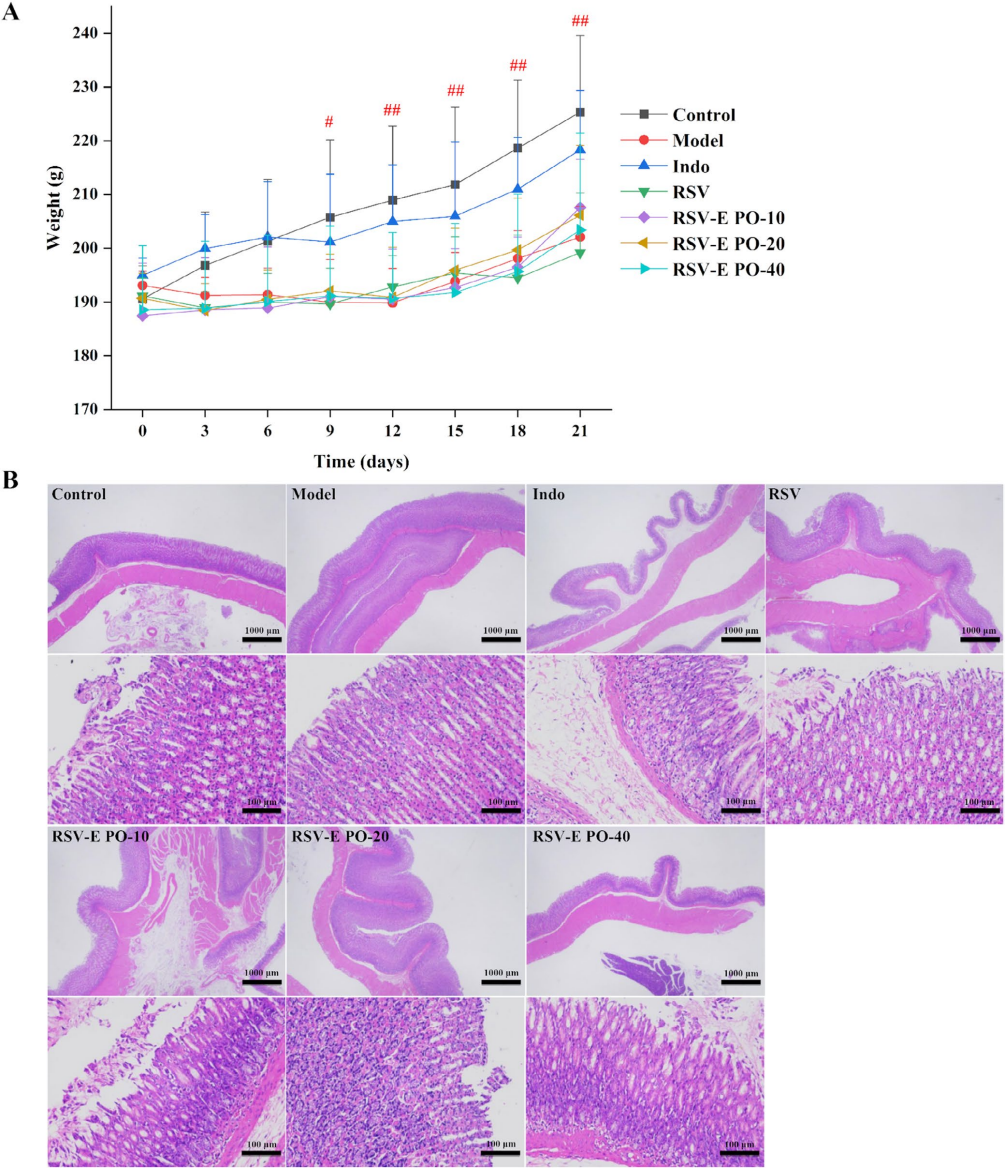
Evaluation of primary safety for RSV‐E PO solid dispersion. (A) Effects of RSV and RSV‐E PO solid dispersion on the body weight in AIA rats, (B) Histopathological analysis of stomach tissues which stained with H&E in adjuvant‐induced arthritis rats. Data are represented as mean ± SD (*n* = 6). #*p* < 0.05, ##*p* < 0.01 vs. control group.

Body weight changes throughout the study were shown in Figure 4A. CFA‐induced arthritis significantly suppressed body weight gain from Day 9 to 21 relative to the control group (*p* < 0.05, *p* < 0.01). Although all pretreatment groups, including those administered RSV and RSV‐E PO solid dispersion, showed a similar trend of weight suppression, their body weights throughout the study remained comparable to the model group, with no statistically significant differences.

No adverse effects or abdominal effusion were noted during the study. The heart, liver, lungs, kidneys, spleen, and thymus were collected for organ index calculation (Table 3). A significant increase in the thymus and spleen indices was observed in the model group compared to the normal group (*p* < 0.05), indicating immune activation. In contrast, all drug intervention groups exhibited a significant reversal of this effect (*p* < 0.05), with organ indices returning to levels comparable to those in the normal group.

**TABLE 3:** Effects of different treatments on organ index of adjuvant‐induced arthritis rats (*n* = 6 in each group).

Organ index (mg/kg)	Control	Model	Indo	RSV	RSV‐E PO‐10	RSV‐E PO‐20	RSV‐E PO‐40
Spleen	1.98 ± 0.10	2.86 ± 1.05^#^	2.35 ± 0.25*	2.23 ± 0.23*	2.27 ± 0.31*	2.05 ± 0.16*	2.06 ± 0.15*
Thymus	1.51 ± 0.29	2.33 ± 0.62^#^	1.31 ± 0.41*	1.45 ± 0.28*	1.71 ± 0.16*	1.60 ± 0.25*	1.60 ± 0.28*
Heart	3.06 ± 0.20	3.06 ± 0.29	3.04 ± 0.24	3.20 ± 0.36	3.09 ± 0.13	3.13 ± 0.27	3.27 ± 0.21
Lung	4.28 ± 0.29	4.63 ± 0.35	4.65 ± 0.73	4.71 ± 0.41	4.33 ± 0.71	4.55 ± 0.49	4.47 ± 0.37
Liver	34.80 ± 3.75	34.38 ± 1.50	37.47 ± 8.00	33.66 ± 2.38	30.75 ± 1.41	31.91 ± 1.92	30.35 ± 1.26
Kidney	7.87 ± 0.44	7.88 ± 0.54	8.36 ± 0.68	8.03 ± 0.87	7.94 ± 0.50	7.88 ± 0.69	7.74 ± 0.39

*Note:* Data = Mean ± SD (*n* = 6). Significance: #*p* < 0.05 vs. control group; *p < 0.05 vs. model group.

#### 3.5.2 Impact of RSV‐E PO Solid Dispersion on Serum Biochemical Parameters

To evaluate the safety of the formulations, serum biomarkers for liver function (ALT, AST) and renal function (CREA, BUN) were analyzed (Table 4). No statistically significant alterations in these key markers were observed in either the model group or any drug‐treated group compared to the normal control. These results demonstrated a favorable safety profile of the RSV‐E PO solid dispersion at the tested doses, indicating an absence of significant hepatorenal damage.

**TABLE 4:** Effects of different treatments on the levels of ALT, AST, creatinine, and blood urea nitrogen in serum of adjuvant‐induced arthritis rats (*n* = 6 in each group).

Group	ALT (U/L)	AST (U/L)	CREA (μmol/L)	BUN (mmol/L)
Control	43.19 ± 2.46	90.29 ± 8.79	25.73 ± 2.90	16.96 ± 1.73
Model	49.21 ± 3.22	97.71 ± 8.09	26.29 ± 3.17	17.15 ± 2.43
Indo	47.93 ± 2.47	102.82 ± 7.26	26.95 ± 2.25	19.41 ± 2.54
RSV	43.27 ± 2.25	104.53 ± 9.15	25.83 ± 1.74	16.32 ± 2.64
RSV‐E PO‐10	44.66 ± 4.54	103.19 ± 9.52	28.85 ± 2.08	20.55 ± 2.73
RSV‐E PO‐20	43.03 ± 3.59	106.97 ± 9.14	28.87 ± 1.06	20.80 ± 2.18
RSV‐E PO‐40	43.28 ± 4.40	105.02 ± 8.84	28.12 ± 2.77	20.83 ± 2.42

*Note:* Data = Mean ± SD (*n* = 6).

#### 3.5.3 Histopathological Impact of RSV‐E PO Solid Dispersion on the Stomach

Histopathological examination of the rat stomach was presented in Figure 4B. The gastric mucosa in the normal, model, RSV, and all RSV‐E PO solid dispersion groups appeared normal, characterized by abundant and well‐organized gastric glands without significant pathological changes. In contrast, the indomethacin group exhibited clear signs of gastric injury, including focal mucosal thinning, detachment of epithelial cells, mild submucosal edema, and loosely arranged connective tissue in the absence of significant inflammatory cell infiltration.

## 4 Discussion

RA is a debilitating autoimmune disease characterized by progressive joint damage and a decline in life quality, with no cure currently available (Gupta et al. 2020). RSV, a natural compound with multi‐faceted pharmacological benefits, has shown promise in alleviating RA progression in preclinical studies. Its therapeutic potential, however, is severely limited by poor bioavailability attributable to its inherent physicochemical limitations. Several studies have reported the preparation of resveratrol (RSV) solid dispersions using hydroxypropyl methylcellulose acetate succinate (HPMCAS) (Fan et al. 2023), magnesium dihydroxide (Spogli et al. 2018), and mesoporous silica microparticles (Li et al. 2016). However, these formulations often suffer from insufficient RSV dissolution efficiency. In the present study, E‐PO not only improved drug wettability and inhibited crystal aggregation but also, more importantly, induced the conversion of RSV to its amorphous state. This amorphous transformation minimized crystal packing energy, thereby conferring a high‐energy thermodynamic state that substantially enhanced both the saturation solubility and dissolution rate of RSV. Given the improved dissolution profile of RSV, its oral bioavailability was consequently enhanced. Accordingly, the resultant increase in systemic exposure was expected to reinforce RSV's well‐documented therapeutic effects in RA.

Comprehensive solid‐state characterization confirmed the amorphous state of RSV in the solid dispersion and its compatibility with E PO. FTIR analysis (Figure 1B) showed no new absorption bands and no shift in the carbonyl peak position, indicating the absence of significant chemical interactions between the components. The amorphous nature of RSV within the solid dispersion was confirmed by DSC and XRD. The DSC thermogram (Figure 1C) displayed the complete disappearance of the crystalline RSV melting endotherm. This finding was corroborated by the XRD pattern (Figure 1D), which exhibited a characteristic amorphous halo. In the physical mixture, the broadening of the RSV melting peak in the DSC thermogram suggested a degree of drug‐polymer miscibility upon heating.

RSV posed a significant challenge for oral delivery due to its poor aqueous solubility (~30 μg·mL^−1^) and inadequate wettability, which collectively result in slow dissolution. This was evidenced by the release of less than 10% of the drug within 2 h (Figure 1A), a profile that severely compromised its bioavailability. The development of formulations to enhance RSV solubility was therefore essential for unlocking its full therapeutic potential. The markedly improved dissolution of the RSV‐E PO solid dispersion could be attributed to several factors facilitated by the polymer. E PO enhanced drug wettability and suppressed crystal aggregation. More importantly, the conversion of resveratrol to its amorphous state minimized crystal packing energy, conferring a high‐energy state that significantly increased saturation solubility and dissolution rate (Jermain et al. 2018). However, the inability to maintain the maximum release over time suggested the occurrence of drug recrystallization from the supersaturated solution. This kinetic profile aligned with the “spring and parachute” model. Here, the amorphous solid dispersion acted as the “spring”, generating a supersaturated state, while the polymer was hypothesized to function as the “parachute” by inhibiting nucleation and crystal growth, thereby temporarily stabilizing the supersaturation and preventing a rapid drop to the crystalline solubility equilibrium (Zi et al. 2019). This behavior was hypothesized to be linked to the polymer's capacity to inhibit drug crystallization in aqueous media.

The oral bioavailability of resveratrol was significantly enhanced by the RSV‐E PO solid dispersion, as evidenced by a 2.08‐fold increase in AUC_(0‐12h)_ compared to raw resveratrol. This improvement was directly attributable to the enhanced in vitro dissolution resulting from the amorphous state of the drug. Interestingly, no significant improvement in *C*_max_ was observed, and *T*_max_ was marginally extended. This pharmacokinetic profile was consistent with the pH‐dependent solubility of E PO, which dissolved primarily in the acidic stomach (pH < 5) (Deng et al. 2021).

Furthermore, the plasma concentration‐time curve of the RSV‐E PO solid dispersion exhibited a distinct double‐peak phenomenon, which might be attributed to several underlying mechanisms. First, raw RSV exhibited a dissolution rate of only ~5% in pH 6.8 medium (Figure S1). In contrast, the RSV‐E PO solid dispersion demonstrated an exceptionally high dissolution rate in the gastric environment (pH ~1–3). As the formulation transited to the small intestine, where the pH is significantly higher, the solubility of E PO decreased markedly, impairing its capacity to sustain drug supersaturation. Consequently, this might lead to drug precipitation, followed by subsequent re‐dissolution and re‐absorption in the intestinal lumen. Additionally, enterohepatic recirculation could be another contributing factor to this atypical absorption pattern (Han et al. 2022; Marier et al. 2002).

The potential of RSV‐E PO solid dispersion prepared in this study for the prevention of RA was performed in a CFA‐induced arthritis rat model. The CFA‐induced arthritis model in rats was a well‐established and widely utilized model for studying RA pathogenesis and therapeutic interventions (Shen et al. 2015). Following model induction, rats developed significant paw swelling, confirming the successful establishment of the RA model. All pretreatment groups exhibited a visible reduction in these pathological features. At the same resveratrol dose (20 mg·kg^−1^), the RSV‐E PO‐20 group showed a significantly greater reduction in paw swelling compared to the raw RSV group. A significant anti‐edema effect was also observed even at the lower dose (RSV‐E PO‐10 group). These results collectively demonstrated that the RSV‐E PO solid dispersion possessed superior anti‐arthritic efficacy relative to raw resveratrol (Figure 2B). In addition, the results of the study indicated that the efficacy of RSV‐E PO solid dispersion enhanced with increasing dose of the solid dispersion.

The pathogenesis of RA involves a complex cascade of immune dysregulation in genetically susceptible individuals, primarily characterized by the aberrant activation of innate and adaptive immunity and a loss of self‐tolerance (Xu et al. 2023). This process is initiated when environmental or pathogenic triggers promote the generation of RA‐related autoantigens, such as citrullinated proteins and heat shock proteins. These autoantigens subsequently activate CD4^+^ T cells, leading to the secretion of pro‐inflammatory cytokines including interferon‐γ (IFN‐γ) and TNF‐α (Zhao et al. 2021). The cytokine milieu then further activates macrophages, monocytes, and synovial fibroblasts, amplifying the inflammatory response through the production of interleukins (e.g., IL‐1β, IL‐6, IL‐15, and IL‐18), which collectively drive synovitis and joint destruction (He et al. 2023). The results of the present study showed a significant dysregulation of serum cytokines in the AIA rat model, characterized by elevated levels of pro‐inflammatory cytokines (IL‐1β, IL‐6, and TNF‐α) and a reduced level of the anti‐inflammatory cytokine IL‐10. Administration of the RSV‐E PO solid dispersion effectively reversed this imbalance, reducing the levels of TNF‐α, IL‐1β, and IL‐6 while elevating IL‐10 levels (Figure 3A–D).

In addition to that, the onset and development of RA have been primarily attributed to the production of oxygen free radicals at the inflamed sites and an elevation in reactive oxygen species (ROS) (Valko et al. 2007). Oxygen free radicals could damage membrane lipids, proteins, DNA, and cartilage, resulting in joint tissue damage in RA (Zamudio‐Cuevas et al. 2022). And the ROS have been implicated in the destruction of cartilage. The successful amelioration of CFA‐induced oxidative damage by the RSV‐E PO solid dispersion (at the middle and high doses), as marked by the normalization of SOD and MDA levels (Figure 3E,F). The current data demonstrated that the limited efficacy of the 10 mg/kg dose indicates that it is likely a marginally effective or sub‐therapeutic dose within the timeframe and severity of our arthritis model. HE staining showed that arthritic rats pretreated with RSV‐E PO solid dispersion exhibited significant decrease in lymphocyte and neutrophil infiltration (Figure 3G).

The RSV‐E PO solid dispersion showed a favorable in vivo safety profile. The stable body weight gain (Figure 4A) and the normalization of organ indices (Table 3) suggested no generalized toxicological effects. Serum biochemical parameters (AST, ALT, CREA, BUN) further confirmed the absence of hepatorenal toxicity (Table 4). The absence of biochemical toxicity was encouraging but that future work will necessitate complete histopathological examination. Finally, the integrity of the gastric mucosa, with no signs of necrosis or significant lesions (Figure 4B), ruled out notable gastrointestinal irritation. These findings supported the conclusion that the solid dispersion formulation is well‐tolerated in vivo.

## 5 Conclusions

In conclusion, the RSV‐E PO solid dispersion was prepared in this study for the enhancement of bioavailability and anti‐arthritis efficacy of RSV due to its high dissolution. Specifically, the bioavailability of resveratrol was increased two‐fold with the solid dispersion, and higher dosages demonstrated enhanced anti‐arthritis in AIA rats. Furthermore, the lack of elevation in serum biomarkers for liver and kidney function suggested a favorable safety profile for this formulation. While these results were compelling, future validation should include long‐term toxicity studies and a control group administered with the polymer alone to fully elucidate its safety and the role of the carrier.

## Author Contributions

**Chungang Zhang:** conceptualization, writing – original draft, writing – review and editing. **Chenchen Yu:** investigation, writing – original draft.

## Funding

This work was supported by the National Natural Science Foundation of China, No. 81503257. Inner Mongolia Major science and technology project, No. 2021ZD0017. Liaoning Provincial Science and Technology Programme Joint Programme (Applied Basic Research Project), 2023JH2/101700206. Liaoning University of Traditional Chinese Medicine‐Natural Science University Key Project, 2021LZY047. Liaoning Provincial Department of Education university basic scientific research project reserve project, LJ212410162055. Changzhi Medical College high‐level talent introduction start‐up fund, GCC202201. Science and technology innovation projects of colleges and universities in Shanxi Province, 2022L374. Liaoning Province Science and Technology Plan Joint Plan (Natural Science Foundation–Doctoral Research Start‐up Project), 2024‐BSLH‐006.

## Ethics Statement

All experimental procedures and housing conditions for animals were in compliance with the Guide of the National Institutes of Health (NIH) for the Care and Use of Laboratory Animals and approved by the Animal Ethics Committee of Liaoning University of Traditional Chinese Medicine (IACUC Issue No. 210000620240226).

## Conflicts of Interest

The authors declare no conflicts of interest.

## Supporting information


**Figure S1:** Dissolution profile of RSV in pH 6.8 (*n* = 3).

## Data Availability

Data will be made available on request.

## References

[fsn371464-bib-0001] Ali, A. , C. Jori , Kanika , et al. 2023. “Recent Trends in Stimuli‐Responsive Hydrogels for the Management of Rheumatoid Arthritis.” Journal of Drug Delivery Science and Technology 89: 104985. 10.1016/j.jddst.2023.104985.

[fsn371464-bib-0002] Baig, M. M. F. A. , L. K. Wong , A. W. Zia , and H. Wu . 2024. “Development of Biomedical Hydrogels for Rheumatoid Arthritis Treatment.” Asian Journal of Pharmaceutical Sciences 19, no. 1: 100887. 10.1016/j.ajps.2024.100887. 38419762 PMC10900807

[fsn371464-bib-0003] Deng, Y. , L. Shen , Y. Yang , and J. Shen . 2021. “Development of Nanoparticle‐Based Orodispersible Palatable Pediatric Formulations.” International Journal of Pharmaceutics 596: 120206. 10.1016/j.ijpharm.2021.120206. 33493595 PMC7980133

[fsn371464-bib-0004] Di Matteo, A. , J. M. Bathon , and P. Emery . 2023. “Rheumatoid Arthritis.” Lancet 402, no. 10416: 2019–2033. 10.1016/S0140-6736(23)01525-8. 38240831

[fsn371464-bib-0005] Fan, W. , J. Wu , M. Gao , X. Zhang , and W. Zhu . 2023. “Preparation of Solid Dispersion of *Polygonum cuspidatum* Extract by Hot Melt Extrusion to Enhance Oral Bioavailability of Resveratrol.” Molecules 28, no. 2: 737. 36677795 10.3390/molecules28020737 PMC9865168

[fsn371464-bib-0006] Gupta, S. , K. P. Mishra , B. Kumar , S. B. Singh , and L. Ganju . 2020. “Andrographolide Attenuates Complete Freund's Adjuvant Induced Arthritis via Suppression of Inflammatory Mediators and Pro‐Inflammatory Cytokines.” Journal of Ethnopharmacology 261: 113022. 10.1016/j.jep.2020.113022. 32569719

[fsn371464-bib-0007] Han, D.‐G. , S.‐W. Seo , E. Choi , et al. 2022. “Impact of Route‐Dependent Phase‐II Gut Metabolism and Enterohepatic Circulation on the Bioavailability and Systemic Disposition of Resveratrol in Rats and Humans: A Comprehensive Whole Body Physiologically‐Based Pharmacokinetic Modeling.” Biomedicine & Pharmacotherapy 151: 113141. 10.1016/j.biopha.2022.113141. 35609369

[fsn371464-bib-0008] He, P. , Y. Que , S. Li , et al. 2023. “Enhanced Anti‐Rheumatic Efficacy of PLGA‐Based Methotrexate‐Loaded Implants in Adjuvant‐Induced Arthritis Rat Model.” Journal of Drug Delivery Science and Technology 88: 104939. 10.1016/j.jddst.2023.104939.

[fsn371464-bib-0009] Hofmann, N. , F. Johann , K. Krollik , et al. 2025. “PROTAC Enabling Formulation In Vivo: Implications of the Polymeric Carrier Eudragit E PO.” Molecular Pharmaceutics 22: 5845–5859. 10.1021/acs.molpharmaceut.5c00303. 40849796 PMC12505254

[fsn371464-bib-0010] Jermain, S. V. , C. Brough , and R. O. Williams 3rd . 2018. “Amorphous Solid Dispersions and Nanocrystal Technologies for Poorly Water‐Soluble Drug Delivery – An Update.” International Journal of Pharmaceutics 535, no. 1–2: 379–392. 10.1016/j.ijpharm.2017.10.051. 29128423

[fsn371464-bib-0011] Khojah, H. M. , S. Ahmed , M. S. Abdel‐Rahman , and E. H. Elhakeim . 2018. “Resveratrol as an Effective Adjuvant Therapy in the Management of Rheumatoid Arthritis: A Clinical Study.” Clinical Rheumatology 37, no. 8: 2035–2042. 10.1007/s10067-018-4080-8. 29611086

[fsn371464-bib-0012] Li, J. , I. W. Lee , G. H. Shin , X. Chen , and H. J. Park . 2015. “Curcumin‐Eudragit E PO Solid Dispersion: A Simple and Potent Method to Solve the Problems of Curcumin.” European Journal of Pharmaceutics and Biopharmaceutics 94: 322–332. 10.1016/j.ejpb.2015.06.002. 26073546

[fsn371464-bib-0013] Li, J. , X. Miao , T. Chen , D. Ouyang , and Y. Zheng . 2016. “Preparation and Characterization of Pelletized Solid Dispersion of Resveratrol With Mesoporous Silica Microparticles to Improve Dissolution by Fluid‐Bed Coating Techniques.” Asian Journal of Pharmaceutical Sciences 11, no. 4: 528–535. 10.1016/j.ajps.2015.10.030.

[fsn371464-bib-0014] Lin, Y. J. , M. Anzaghe , and S. Schülke . 2020. “Update on the Pathomechanism, Diagnosis, and Treatment Options for Rheumatoid Arthritis.” Cells 9, no. 4: 880. 10.3390/cells9040880. 32260219 PMC7226834

[fsn371464-bib-0015] Mahdi, H. J. , N. A. K. Khan , M. Z. B. Asmawi , R. Mahmud , and V. Murugaiyah . 2018. “In Vivo Anti‐Arthritic and Anti‐Nociceptive Effects of Ethanol Extract of *Moringa oleifera* Leaves on Complete Freund's Adjuvant (CFA)‐Induced Arthritis in Rats.” Integrative Medicine Research 7, no. 1: 85–94. 10.1016/j.imr.2017.11.002. 29629295 PMC5884001

[fsn371464-bib-0016] Marier, J.‐F. , P. Vachon , A. Gritsas , J. Zhang , J. P. Moreau , and M. P. Ducharme . 2002. “Metabolism and Disposition of Resveratrol in Rats: Extent of Absorption, Glucuronidation, and Enterohepatic Recirculation Evidenced by a Linked‐Rat Model.” Journal of Pharmacology and Experimental Therapeutics 302, no. 1: 369–373. 10.1124/jpet.102.033340. 12065739

[fsn371464-bib-0017] Moseson, D. E. , T. B. Tran , B. Karunakaran , R. Ambardekar , and T. N. Hiew . 2024. “Trends in Amorphous Solid Dispersion Drug Products Approved by the U.S. Food and Drug Administration Between 2012 and 2023.” International Journal of Pharmaceutics: X 7: 100259. 10.1016/j.ijpx.2024.100259. 38974024 PMC11225173

[fsn371464-bib-0018] Peng, Y. , Y. Huang , H. Li , et al. 2024. “Associations Between Rheumatoid Arthritis and Intestinal Flora, With Special Emphasis on RA Pathologic Mechanisms to Treatment Strategies.” Microbial Pathogenesis 188: 106563. 10.1016/j.micpath.2024.106563. 38331355

[fsn371464-bib-0019] Refaat, R. , M. Salama , E. Abdel Meguid , A. El Sarha , and M. Gowayed . 2013. “Evaluation of the Effect of Losartan and Methotrexate Combined Therapy in Adjuvant‐Induced Arthritis in Rats.” European Journal of Pharmacology 698, no. 1: 421–428. 10.1016/j.ejphar.2012.10.024. 23117086

[fsn371464-bib-0020] Shen, A.‐Z. , X. Li , W. Hu , and F. H. Chen . 2015. “Total Flavonoids of *Bidens bipinnata* L. Ameliorate Experimental Adjuvant‐Induced Arthritis Through Induction of Synovial Apoptosis.” BMC Complementary and Alternative Medicine 15, no. 1: 437. 10.1186/s12906-015-0962-3. 26669668 PMC4681046

[fsn371464-bib-0021] Spogli, R. , M. Bastianini , F. Ragonese , et al. 2018. “Solid Dispersion of Resveratrol Supported on Magnesium DiHydroxide (Resv@MDH) Microparticles Improves Oral Bioavailability.” Nutrients 10, no. 12: 1925. 30563110 10.3390/nu10121925 PMC6315708

[fsn371464-bib-0022] Tian, B. , and J. Liu . 2020. “Resveratrol: A Review of Plant Sources, Synthesis, Stability, Modification and Food Application.” Journal of the Science of Food and Agriculture 100, no. 4: 1392–1404. 10.1002/jsfa.10152. 31756276

[fsn371464-bib-0023] Valko, M. , D. Leibfritz , J. Moncol , M. T. Cronin , M. Mazur , and J. Telser . 2007. “Free Radicals and Antioxidants in Normal Physiological Functions and Human Disease.” International Journal of Biochemistry & Cell Biology 39, no. 1: 44–84. 10.1016/j.biocel.2006.07.001. 16978905

[fsn371464-bib-0024] Wang, T. , G. Wang , Y. Zhang , J. Zhang , W. Cao , and X. Chen . 2019. “Effect of Lentivirus‐Mediated Overexpression or Silencing of MnSOD on Apoptosis of Resveratrol‐Treated Fibroblast‐Like Synoviocytes in Rheumatoid Arthritis.” European Journal of Pharmacology 844: 65–72. 10.1016/j.ejphar.2018.12.001. 30529106

[fsn371464-bib-0025] Xu, Y. , M. Zhao , J. Cao , et al. 2023. “Applications and Recent Advances in Transdermal Drug Delivery Systems for the Treatment of Rheumatoid Arthritis.” Acta Pharmaceutica Sinica B 13, no. 11: 4417–4441. 10.1016/j.apsb.2023.05.025. 37969725 PMC10638506

[fsn371464-bib-0026] Yang, G. , C.‐C. Chang , Y. Yang , et al. 2018. “Resveratrol Alleviates Rheumatoid Arthritis via Reducing ROS and Inflammation, Inhibiting MAPK Signaling Pathways, and Suppressing Angiogenesis.” Journal of Agricultural and Food Chemistry 66, no. 49: 12953–12960. 10.1021/acs.jafc.8b05047. 30511573

[fsn371464-bib-0027] Zamudio‐Cuevas, Y. , K. Martínez‐Flores , G. A. Martínez‐Nava , et al. 2022. “Rheumatoid Arthritis and Oxidative Stress.” Cellular and Molecular Biology (Noisy‐le‐Grand, France) 68, no. 6: 174–184. 10.14715/cmb/2022.68.6.28. 36227658

[fsn371464-bib-0028] Zewail, M. , N. Nafee , M. W. Helmy , and N. Boraie . 2019. “Coated Nanostructured Lipid Carriers Targeting the Joints – An Effective and Safe Approach for the Oral Management of Rheumatoid Arthritis.” International Journal of Pharmaceutics 567: 118447. 10.1016/j.ijpharm.2019.118447. 31226475

[fsn371464-bib-0029] Zhao, J. , X. Chen , K.‐H. Ho , et al. 2021. “Nanotechnology for Diagnosis and Therapy of Rheumatoid Arthritis: Evolution Towards Theranostic Approaches.” Chinese Chemical Letters 32, no. 1: 66–86. 10.1016/j.cclet.2020.11.048.

[fsn371464-bib-0030] Zi, P. , C. Zhang , C. Ju , et al. 2019. “Solubility and Bioavailability Enhancement Study of Lopinavir Solid Dispersion Matrixed With a Polymeric Surfactant – Soluplus.” European Journal of Pharmaceutical Sciences 134: 233–245. 10.1016/j.ejps.2019.04.022. 31028820

