# Supplementary figures and images for: High Bioavailability Resveratrol Delivery System: A Novel Nutritional Strategy for the Prevention and Alleviation of Rheumatoid Arthritis
Source: Food Sci Nutr. 2026 Feb 4;14(2):e71464. doi: 10.1002/fsn3.71464 (PMC12872117; doi:10.1002/fsn3.71464)

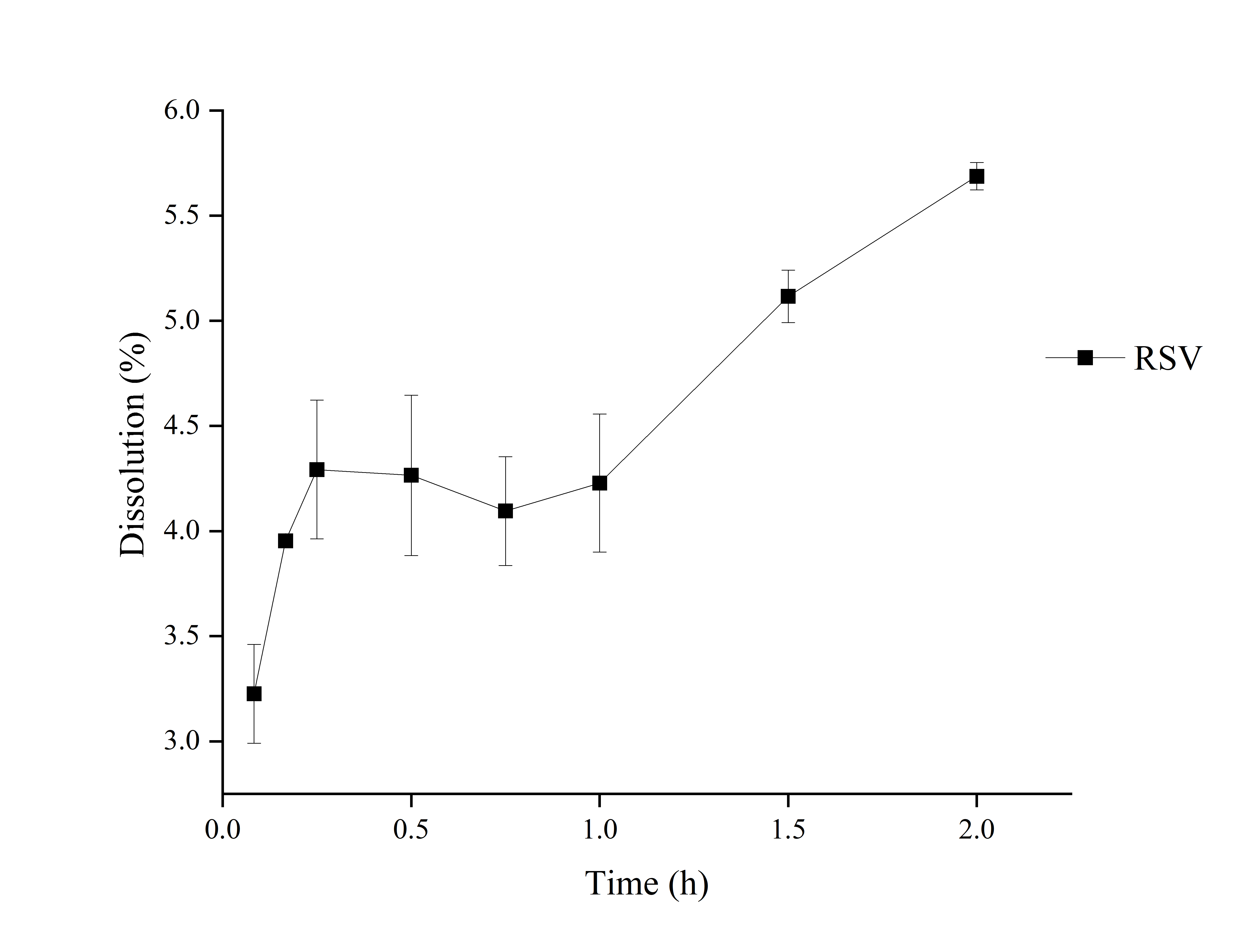


Figure S1. Dissolution profile of RSV in pH 6.8 (n=3).

Supplement: Supplementary file 1 — Figure S1: Dissolution profile of RSV in pH 6.8 (n = 3). [file FSN3-14-e71464-s001.docx]
